# Ammonium fluoride-activated synthesis of cubic *δ*-TaN nanoparticles at low temperatures

**DOI:** 10.1186/1556-276X-8-126

**Published:** 2013-03-15

**Authors:** Young-Jun Lee, Dae-Young Kim, Kap-Ho Lee, Moon-Hee Han, Kyoung-Soo Kang, Ki-Kwang Bae, Jong-Hyeon Lee

**Affiliations:** 1 Graduate School of Green Energy Technology, Chungnam National University, Daejeon 305-764, Republic of Korea; 2 Graduate School of Department of Metallurgical Engineering, Chungnam National University, Daejeon 305-764, Republic of Korea; 3 Korea Institute of Energy Research, 152 Gajeong-ro, Yuseong-gu, Daejeon 305-343, Republic of Korea

**Keywords:** Cubic *δ*-TaN, Combustion synthesis, Ammonium fluoride, Nanoparticles

## Abstract

Cubic delta-tantalum nitride (*δ*-TaN) nanoparticles were selectively prepared using a K_2_TaF_7_ + (5 + *k*) NaN_3_ + *k*NH_4_F reactive mixture (*k* being the number of moles of NH_4_F) via a combustion process under a nitrogen pressure of 2.0 MPa. The combustion temperature, when plotted as a function of the number of moles of NH_4_F used, was in the range of 850°C to 1,170°C. X-ray diffraction patterns revealed the formation of cubic *δ*-TaN nanoparticles at 850°C to 950°C when NH_4_F is used in an amount of 2.0 mol (or greater) in the combustion experiment. Phase pure cubic *δ*-TaN synthesized at *k* = 4 exhibited a specific surface area of 30.59 m^2^/g and grain size of 5 to 10 nm, as estimated from the transmission electron microscopy micrograph. The role of NH_4_F in the formation process of *δ*-TaN is discussed with regard to a hypothetical reaction mechanism.

## Background

Among the various transition-metal nitrides, TaN is a material that has potential for application in microelectronic components such as capacitors, thin-film resistors, and barrier materials that prevent the diffusion of copper into silicon [1,2]. In addition, TaN has been used in high-temperature ceramic pressure sensors because of its good piezoresistive properties [3]. Also, it is an attractive histocompatible material that can be used in artificial heart valves [4]. Among the various tantalum nitride phases, cubic delta-tantalum nitride (*δ*-TaN), with a NaCl-type structure (space group: Fm3m), exhibits excellent properties such as high hardness, stability at high temperature, and superconductivity [5].

In general, it is difficult to produce *δ*-TaN under ambient conditions since its formation requires high temperature and nitrogen pressure. According to the data reported in another study [6], *δ*-TaN is normally made at more than 1,600°C and 16 MPa of nitrogen pressure. Kieffer et al. synthesized cubic TaN by heating hexagonal TaN above 1,700°C at a N_2_ pressure of 6 atm [7]. Matsumoto and Konuma were successful in producing cubic TaN by heating hexagonal TaN at a reduced pressure using a plasma jet [8]. Mashimo et al. were able to transform hexagonal TaN into cubic TaN by both static compression and shock compression at high temperature [9]. Cubic TaN in powder form was also synthesized by self-propagating high-temperature synthesis technique [10,11]. In this process, the combustion of metallic tantalum from 350 to 400 MPa of nitrogen pressure resulted in micrometer size *δ*-TaN at a temperature above 2,000°C.

More recently, two approaches, solid-state metathesis reaction and nitridation-thermal decomposition [12-14], were adopted for the synthesis of nanosized particles of *δ*-TaN. O’Loughlin et al. used the metathesis reaction of TaCl_5_ with Li_3_N and 12 mol of NaN_3_ to produce *δ*-TaN [12]. The authors concluded that significant nitrogen pressure created by the addition of NaN_3_ enabled cubic-phase TaN to form, along with hexagonal Ta_2_N. Solid-state metathesis reaction applied to the TaCl_5_-Na-NH_4_Cl mixture resulted in a bi-phase product at 650°C comprising both hexagonal and cubic phases of TaN [13]. More recently, Liu et al. reported the synthesis of cubic *δ*-TaN through homogenous reduction of TaCl_5_ with sodium in liquid ammonia, with a subsequent annealing process at 1,200°C to 1,400°C under high vacuum [14]. Nitridation-thermal decomposition, a two-step process for the synthesis of cubic *δ*-TaN, was also reported [15]. In the first step, nanosized Ta_2_O_5_ was nitrided at 800°C for 8 h under an ammonia flow. The as-prepared product was then thermally decomposed at 1,000°C in nitrogen atmosphere, and cubic nanocrystalline *δ*-TaN was obtained.

In most cases, the products prepared by the above-mentioned methods were often mixtures containing other compounds such as TaN_0.5_ or other nonstoichiometric phases. Therefore, synthesis inefficiency of cubic *δ*-TaN nanoparticles by known approaches coupled with the multiphase composition of products makes this topic challenging and scientifically attractive.

In this paper, an attractive and rapid approach for synthesizing cubic *δ*-TaN nanoparticles is developed. This approach includes the combustion of K_2_TaF_7_ + (5 + *k*)NaN_3_ + *k*NH_4_F exothermic mixture under nitrogen atmosphere and water purification of final products to produce cubic *δ*-TaN. The approach described in this study is simple and cost-effective for the large-scale production of *δ*-TaN.

## Methods

For sample preparation, the following chemicals were used: K_2_TaF_7_ (prepared at the Graduate School of Green Energy Technology, Chungnam National University, Korea), NaN_3_ powder (99.0% purity; particle size < 50 μm; Daejung Chemical and Metals Co., Ltd., Shiheung City, South Korea). Chemical-grade ammonium halides (NH_4_F and NH_4_Cl) were purchased from Samchun Pure Chemical Co., Ltd., Pyeongtaek City, South Korea. All salts were handled in a glove box in dry argon atmosphere (99.99%; Messer, Northumberland, UK).

**Figure 1:**
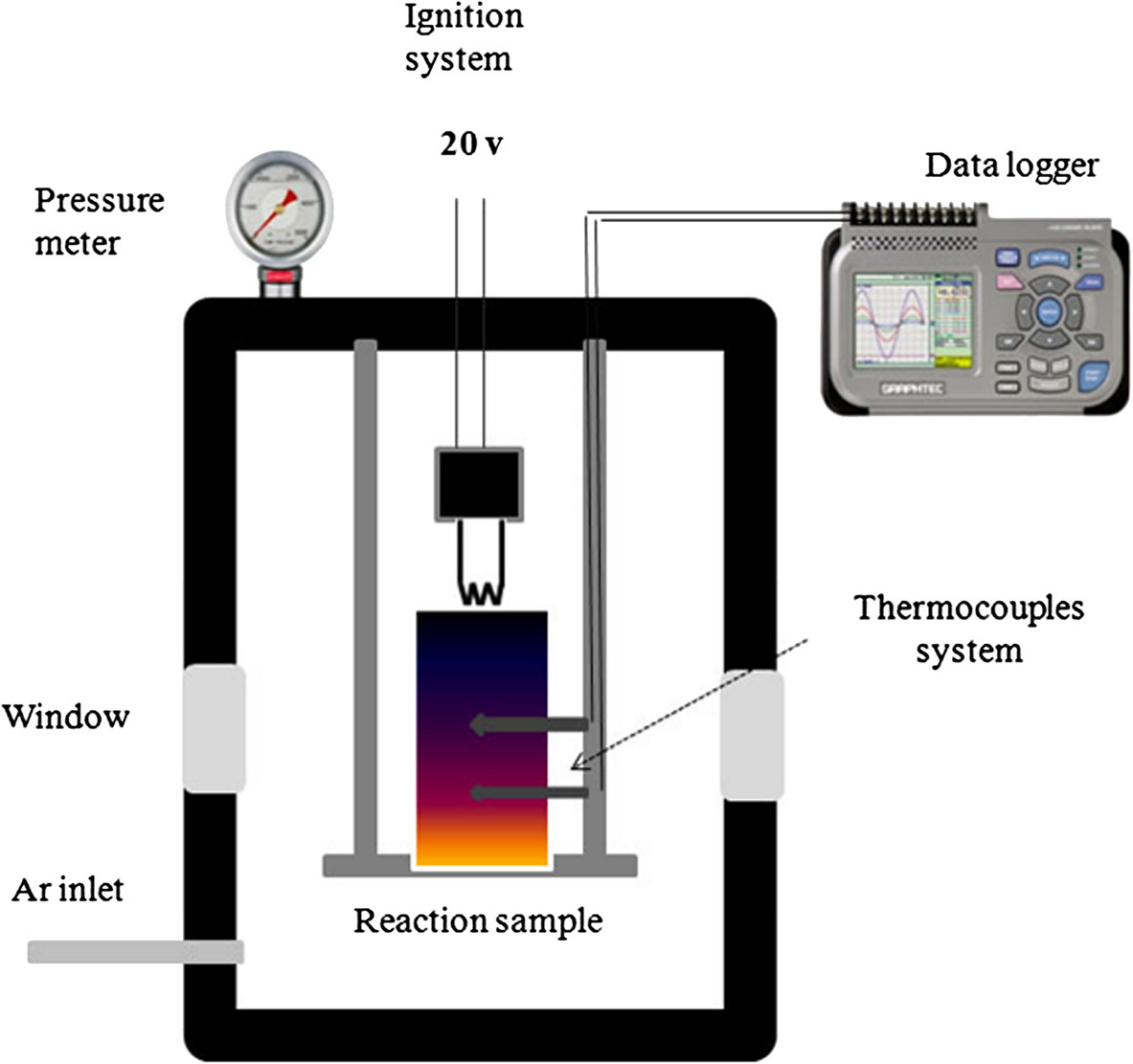
Experimental setup for the synthesis cubic TaN nanoparticles.

To prepare the reaction mixture, controlled amounts of reactant powders, K_2_TaF_7_, NaN_3_, and NH_4_F, were weighed and thoroughly mixed in a glove box in argon atmosphere. About 60 to 80 g of the mixture was compacted by hand in a stainless steel cup (4.0 cm in diameter) and placed in a high-pressure reaction vessel for combustion (Figure 1). A vacuum was applied to remove the air from the combustion vessel, which was then filled with nitrogen gas with a pressure of 2.0 MPa. The combustion process was initiated by a hot nickel-chromium filament system, and the reaction temperatures were measured using WR-20/WR-5 thermocouples inserted into the reaction pellet. After completion of the combustion process, the burned-down sample was cooled to room temperature and transferred to a 500-ml beaker for further purification. The sample was purified by washing with distilled water in order to remove the NaF and KF salts that formed during the reaction. The purified black powder was dried in air at 80°C to 90°C.

We used the simulation software ‘Thermo’ to predict adiabatic combustion temperature (*T*_ad_) and concentrations of equilibrium phases in the combustion wave [16]. Calculations of equilibrium characteristics were based on minimizing the thermodynamic potential of the system. The initial parameters (temperature and pressure) of the system were set as 25°C and 2.0 MPa, respectively.

The crystal structure and morphology of the TaN nanoparticles were characterized X-ray diffraction (XRD) with Cu Kα radiation (D5000, Siemens AG, Munich, Germany), field-emission scanning electron microscopy (FESEM; JSM 6330F, JEOL Ltd., Akishima, Tokyo, Japan), and transmission electron microscopy (TEM; JEM 2010, JEOL Ltd.). The specific surface area of the nanoparticles was determined from the linear portion of the Brunauer, Emmett, and Teller plot.

## Results and discussion

### Results

#### Adiabatic combustion temperature and equilibrium phases

**Figure 2:**
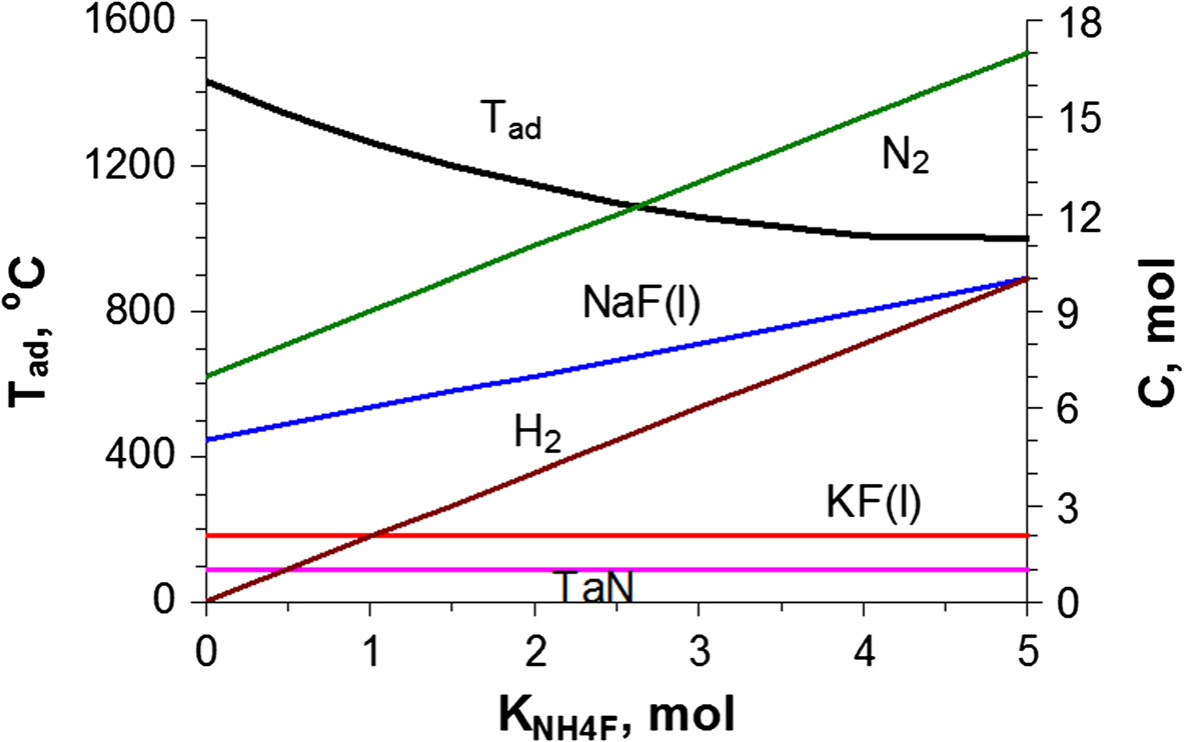
***T***_**ad **_**and equilibrium phases in K**_**2**_**TaF**_**7 **_**+ (5 + *k*)NaN**_**3 **_**+ *k*NH**_**4**_**F system upon *k*.**

The combustion thermodynamics of the K_2_TaF_7_ + (5 + *k*)NaN_3_ + *k*NH_4_F system calculated by the Thermo simulation software is shown Figure 2. This calculation provides equilibrium product concentration (C) and *T*_ad_ as a function of the number of moles of NH_4_F used (*k*). As shown in Figure 2, the calculated adiabatic combustion temperature shows an almost linear decreasing tendency with increasing *k*. The mixture with the highest temperature, near 1,425°C, is predicted for K_2_TaF_7_ + 5NaN_3_ binary mixture (*k* = 0). As estimated from Figure 2, the temperature change from 1,425°C to 1,000°C is observed when *k* changes from 0 to 5. The reaction products predicted by thermodynamic analysis comprise solid tantalum nitride (TaN), liquid fluorides of alkaline metal (NaF, KF), and gaseous H_2_ and N_2_. The concentration of TaN and KF predicted by thermodynamic analysis is constant in the given interval of NH_4_F, whereas the concentration of NaF, H_2_, and N_2_ has been increasing with increasing *k*. Intensive gas release in the designed system, especially at higher *k*, may generate high pressure in the combustion wave. Our estimation shows that the pressure in the combustion wave may reach tens and even hundreds of atmospheres. This can be very helpful to accelerate the formation of cubic phase TaN at given temperatures. This also indicates that one must keep external nitrogen pressure relatively high to prevent distortion of the sample during the combustion experiment and to avoid the scattering of reaction mass inside of the combustion chamber. Therefore, the data obtained from thermodynamic analysis can serve as a good theoretical guideline for controlling the combustion process and optimizing the synthesis conditions of cubic TaN nanoparticles.

#### DSC-TGA curves and combustion parameters

**Figure 3:**
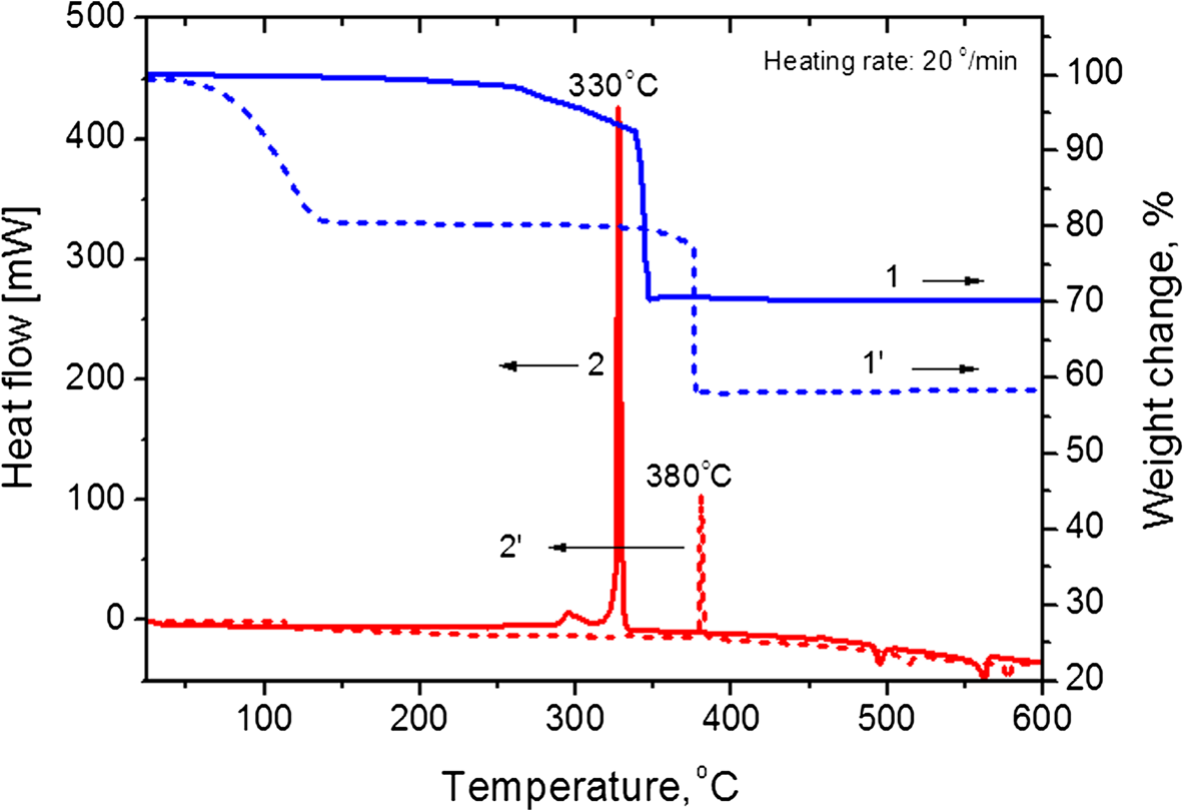
**DSC-TGA curves of K**_**2**_**TaF**_**7 **_**+ 5NaN**_**3 **_**and K**_**2**_**TaF**_**7 **_**+ 9NaN**_**3 **_**+ 4NH**_**4**_**F systems in argon atmosphere.**

Differential scanning calorimetry (DSC) and thermogravimetric analysis (TGA) were carried out in order to elucidate the thermal behavior of the K_2_TaF_7_ + 5NaN_3_ (C1) and K_2_TaF_7_ + 5NaN_3_ + 4NH_4_F (C2) reaction mixtures as well as to determine the weight losses incurred during the heating process. The samples were heated at a rate of 20°C/min in a flow of argon gas. The weight loss for both samples is in the range from approximately 60°C to 380°C (Figure 3, lines 1 and 1^′^) which is mainly caused by the decomposition of NH_4_F and NaN_3_. Therefore, above 380°C, no drop of mass was recorded by TGA analysis. The highest maximum of DSC signals (Figure 3, lines 2 and 2^′^) is reached at 330°C and 380°C. This means that at the given temperatures, a strong exothermic reduction of K_2_TaF_7_ by Na has occurred in the C1 and C2 mixtures, resulting in large outflow of heat and sharp weight losses. In addition, the exothermic peak at round 330°C (mixture C1) is significantly higher than the exothermic peak recorded at around 380°C for C2. This indicates that the NH_4_F-containing mixture is less exothermic, and this finding is concise with the thermodynamic analysis data. Therefore, it can be suggested that in the given systems, the combustion process in the K_2_TaF_7_ + (5 + *k*)NaN_3_ + *k*NH_4_F system starts at around 350 ± 50°C.

**Figure 4:**
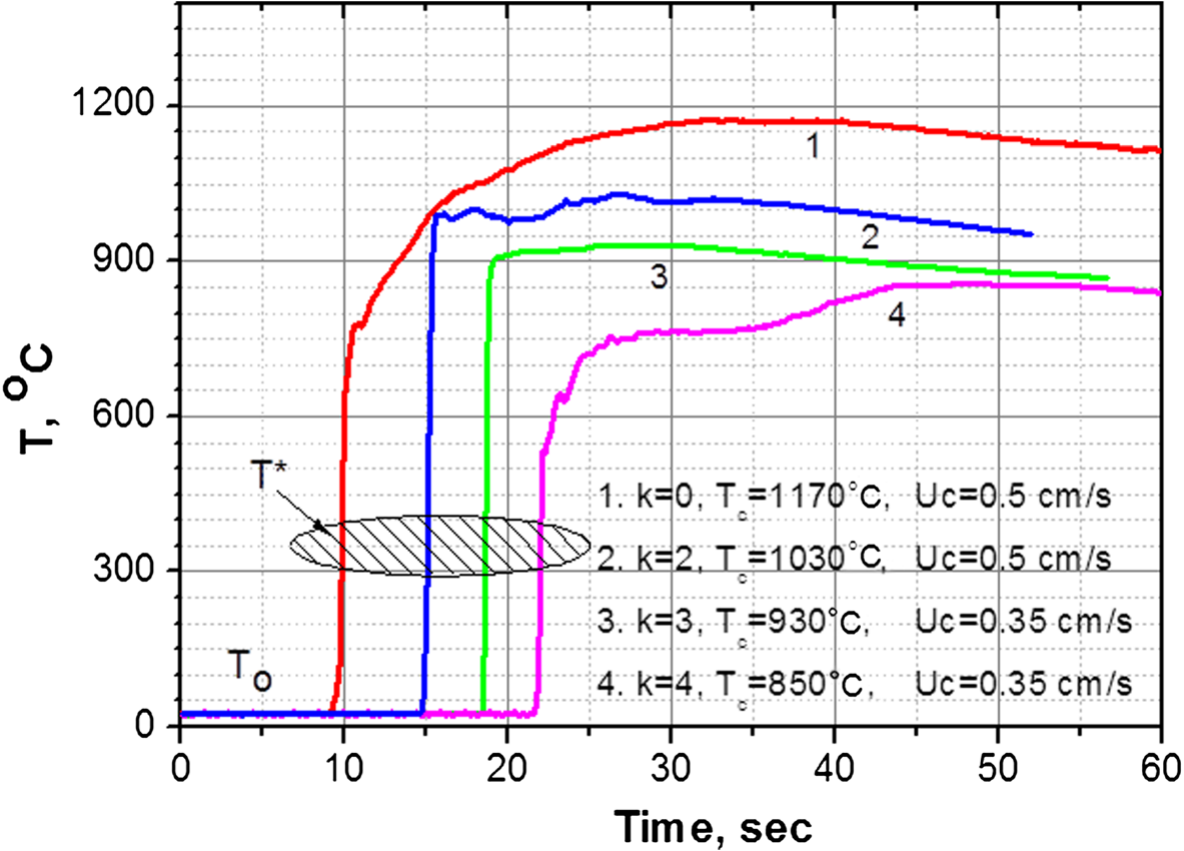
**Temperature-time profiles in K**_**2**_**TaF**_**7 **_**+ (5 + *k*)NaN**_**3 **_**+ *k*NH**_**4**_**F system.**

Figure 4 shows the temperature-time profiles for the combustion wave of the K_2_TaF_7_ + (5 + *k*)NaN_3_ + *k*NH_4_F mixture over the reaction time (*t*). As shown in Figure 4, the starting temperature for the combustion process is denoted by T* (350 ± 50°C) and corresponds to the sharp peaks in the DSC curve (Figure 3). One can see that in the beginning of the reaction zone, the temperature increases rapidly from 25°C to 700°C and then to 1,000°C, and then long-tailed post-combustion processes followed. The combustion temperature (*T*_c_) showed a tendency to decrease with the amount of NH_4_F used. In the investigated interval of *k*, the *T*_c_ drops from 1,170°C (*k* = 0) to 850°C (*k* = 4). The maximum combustion velocity (*U*_c_ = 0.5 cm/s) occurred at the nearly stoichiometric mixture (*k* = 0), but combustion velocity decreased significantly as the K_2_TaF_7_ + 5NaN_3_ mixture became ‘diluted’ with NH_4_F.

#### Characteristics of combusted samples and powders

**Figure 5:**
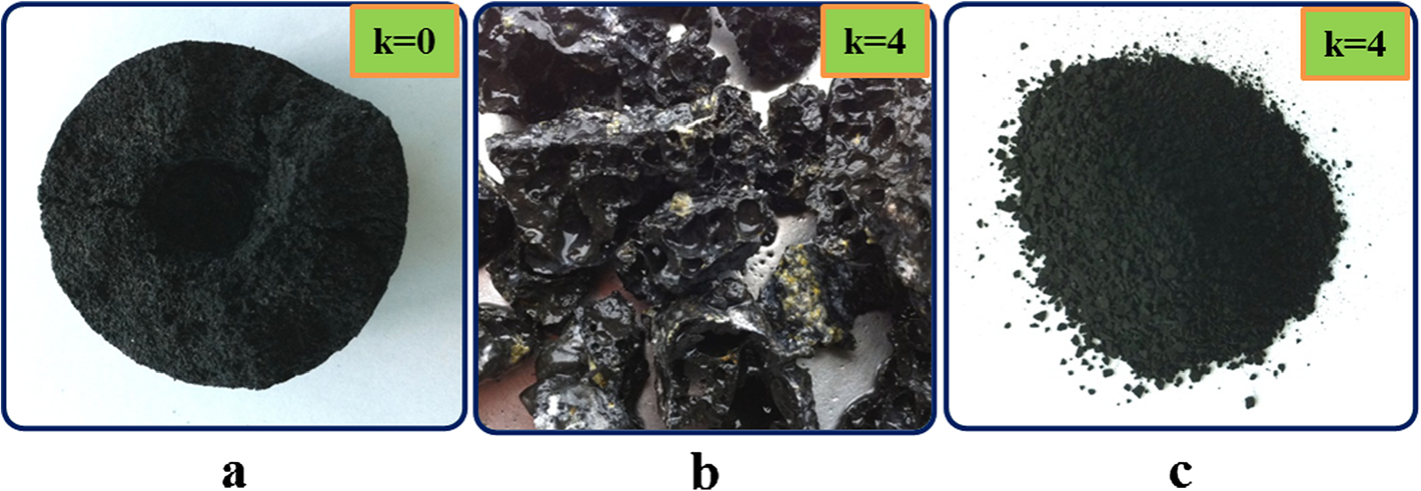
Photographs of as-combusted (a, b) and water-purified (c) samples.

Figure 5 shows photographs of the as-combusted (Figure 5a,b) and water-purified (Figure 5c) samples. After combustion, the sample of the K_2_TaF_7_ + 5NaN_3_ composition (*k* = 0) retained its original shape and size (Figure 5a). However, the samples produced using 2.0 to 4.0 mol of NH_4_F had melted after the combustion process, forming a brown-colored, brittle, and shapeless molten product. For instance, several fragments of the sample prepared with *k* = 4 are shown in Figure 5b. Many large pores, due to the release of N_2_ and H_2_ gases during the combustion process, can be seen in the solid molten mass. After dissolving alkali fluorides (NaF and KF) into warm distillated water, TaN fine powders were obtained. A photograph of finally purified TaN samples prepared from the K_2_TaF_7_ + 5NaN_3_ +4NH_4_F mixture is shown in Figure 5c. Its color is uniformly black, and specific gravity lies between 0.7 and 0.9 g/cm^3^.

**Figure 6:**
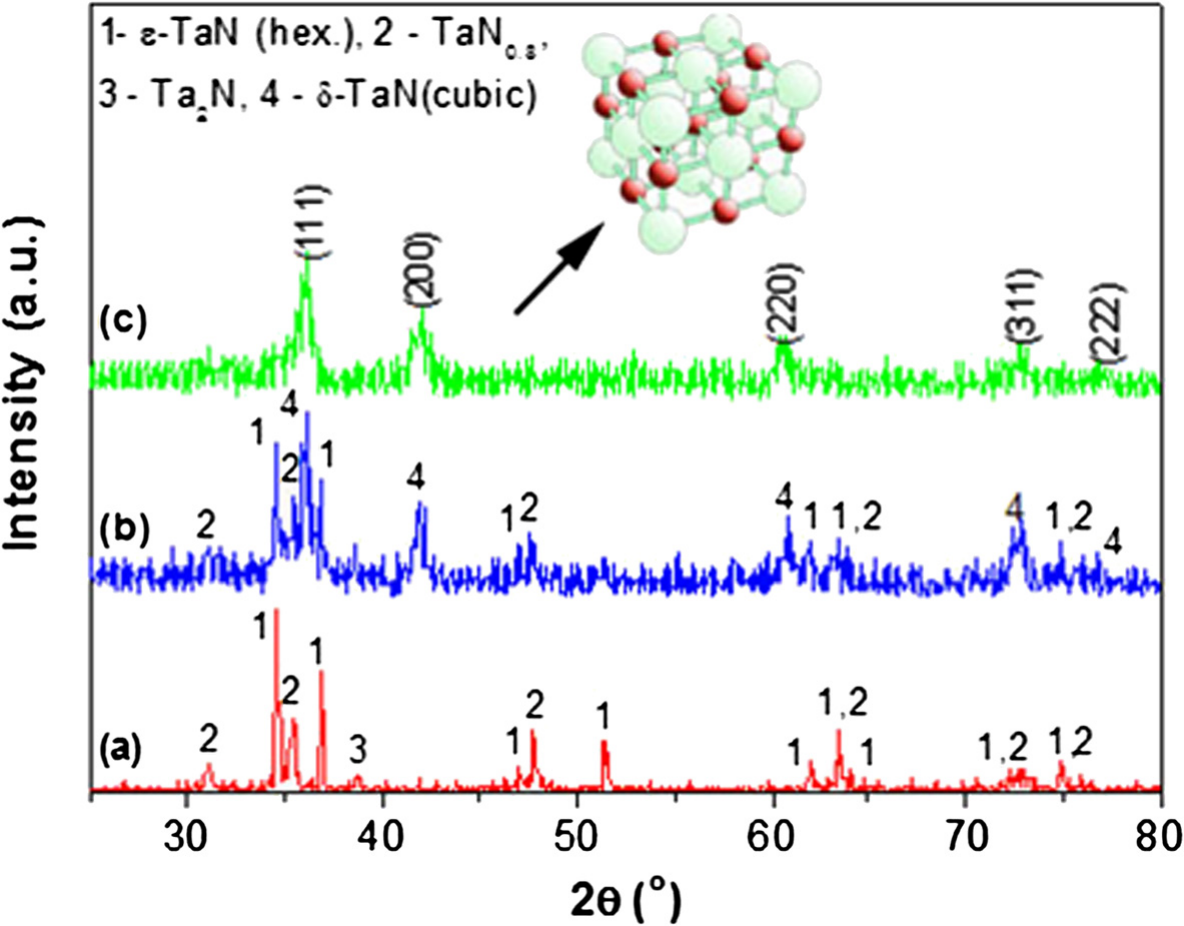
**XRD patterns of water-purified powders synthesized from K**_**2**_**TaF**_**7 **_**+ (5 + *k*)NaN**_**3 **_**+ *k*NH**_**4**_**F mixture.** (**a**) *k* = 0, (**b**) *k* = 2.0, and (**c**) *k* = 4.0.

The XRD patterns for the water-purified powders that had been prepared with different amounts of NH_4_F are shown in Figure 6. Diffraction peaks of the sample prepared at *k* = 0 (without NH_4_F) indicate three nitride phases: hexagonal *ε*-TaN, TaN_0.8_, and Ta_2_N (Figure 6a). The cubic *δ*-TaN phase was detected in large amounts, along with the *ε*-TaN and TaN_0.8_ phases for samples with *k* = 2 (Figure 6b). By applying 4 mol of NH_4_F to the reaction of K_2_TaF_7_ and NaN_3_, the only crystalline product produced is cubic TaN. The diffraction peaks marked in Figure 6c correspond to face-centered cubic TaN (JCPDS 32–1283). Thus, the reaction of K_2_TaF_7_, NaN_3_, and NH_4_ (in a 1:9:4 molar ratio) results in the formation of phase-pure crystalline cubic TaN. The peaks for *δ*-TaN are weak and broad, indicating the small size of its particles. The lattice parameter calculated from the highest intensity peak (111) was *a* = 4.32 Å. This was in good agreement with the previously reported value of 0.433 ± 0.001 nm for thin films [17]. The nitrogen content in the powders at various *k* values is shown in Table 1. It shows that the nitrogen content at *k* = 0 is 7.01%, which corresponds to the TaN_0.98_ composition. With increasing *k*, the nitrogen content then slowly drops down, reaching to 6.51% at *k* = 4. This amount of nitrogen theoretically corresponds to the TaN_0.91_ composition. All the powders contain about 0.15% carbon.

**Table 1:** Content of nitrogen in TaN

***k *(mol)**	**N (%)**	**Formula**
0	7.01	TaN_0.98_
2	6.95	TaN_0.97_
3	6.72	TaN_0.94_
4	6.51	TaN_0.91_

**Figure 7:**
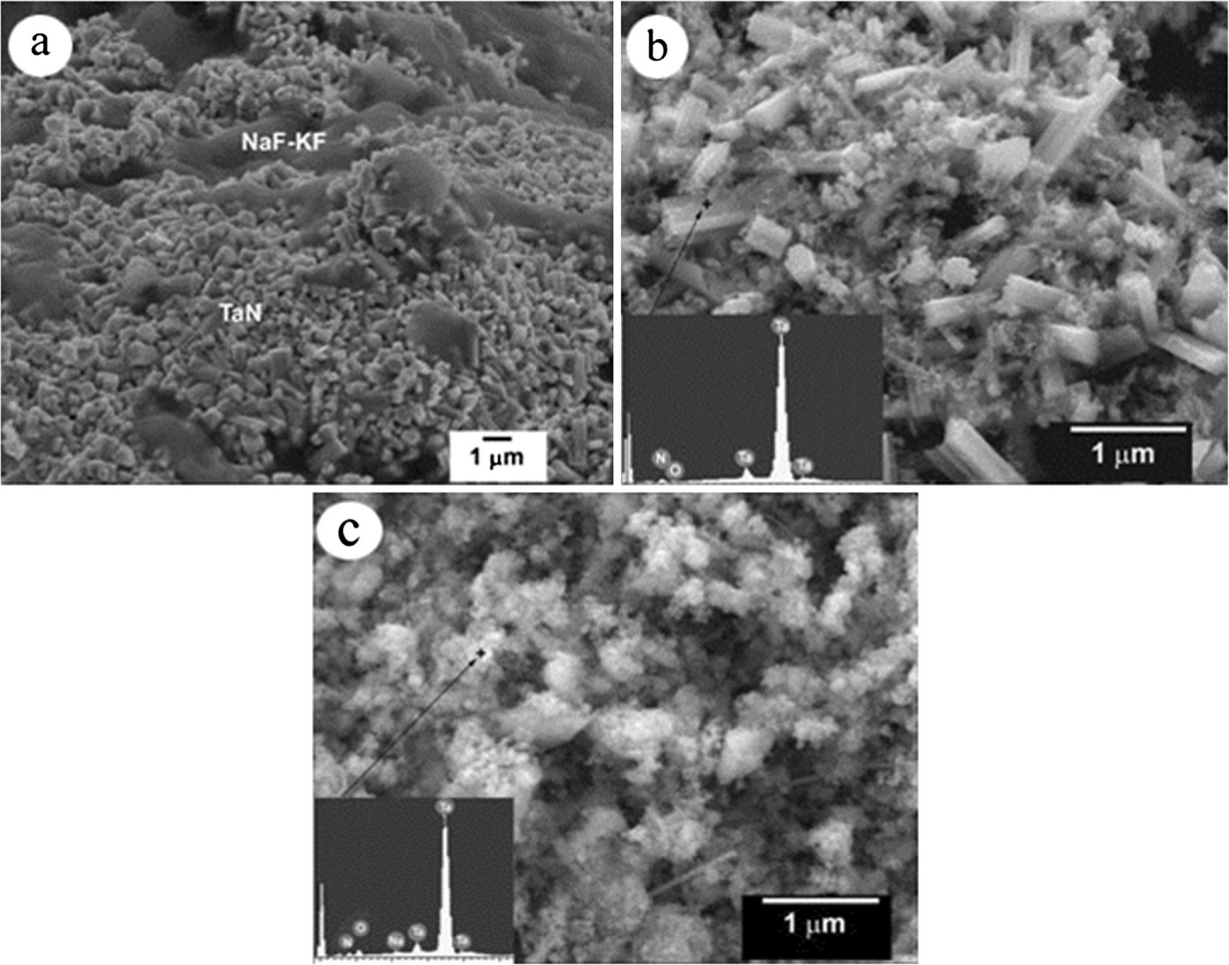
**SEM micrographs of reaction product (a), and water-purified TaN samples with EDX analysis (b, c).** (**a**) *k* = 0, (**b**) *k* = 0, and (**c**) *k* = 4.

The SEM microstructure of the combustion product (*k* = 0) right after the synthesis process is shown in Figure 7a. Due to a large portion of molten fluorides (5NaF to 2KF), the final product has a molten microstructure in which the crystalline particles of tantalum nitride are embedded. The microstructure of the same sample after water purification is shown in Figure 7b. A part of TaN particles were crystallized in a rodlike fashion; at that, the length of rods is about 0.5 to 1.5 μm, as estimated from the micrograph. A large portion of small particles whose sizes are on the order of submicrometers also exist on the same micrograph. We think that the presence of different-sized particles in Figure 7b can be associated with the phase composition of the product, i.e., the rod-shaped particles most likely are those of hexagonal *ε*-TaN, whereas the small-sized particles belong to the TaN_0.8_ and Ta_2_N phases. With an increase in *k*, the rod-shaped particles disappeared, and the size of particles became smaller and uniform. As a typical example, the micrograph of the cubic *δ*-TaN particles produced using 4.0 mol of NH_4_F is shown in Figure 7c. These particles are less than 100 nm in size. They usually exist in the form of relatively large clusters (0.5 to 1.0 μm), owing to the attractive forces between the particles. EDS analysis taken from rodlike and small-sized particles (Figure 7b,c) shows Ta and N as the main elements; however, small peaks of oxygen also exist.

**Figure 8:**
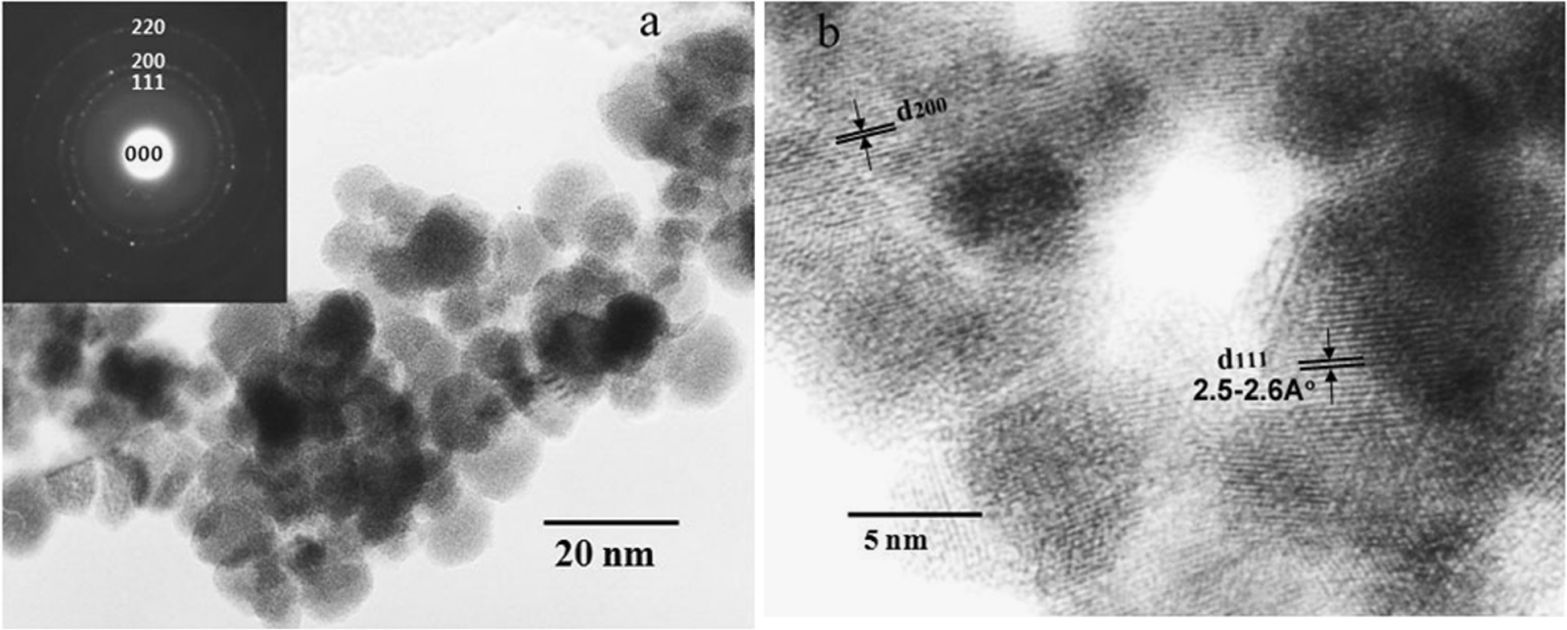
TEM micrograph (a), SAED pattern (inset of a), and HRTEM image (b) of cubic TaN nanoparticles.

Figure 8a shows the TEM image and the corresponding selected area electron diffraction (SAED) pattern of the cubic *δ*-TaN nanoparticles synthesized at 800°C from the K_2_TaF_7_ + 9NaN_3_ + 4NH_4_F mixture. The TEM image confirmed the formation of TaN nanoparticles, which had an average size of <10 nm. From the diffraction rings in the SAED pattern, shown in the inset of Figure 8a, (111), (200), and (220) planes were identified in the *δ*-TaN nanoparticles. The average spacing between the stacks was 2.5 to 2.6 Å (111), as estimated from the HRTEM image (Figure 7b).

## Discussion

The phase-pure cubic TaN nanoparticles reported here have proven to be difficult to synthesize in previous attempts using solid-state metathesis reactions [12-14]. However, our experimental results clearly indicate that cubic-phase *δ*-TaN nanoparticles can be produced at moderate temperatures, within several or tens of seconds by combustion of the K_2_TaF_7_ + (5 + *k*) NaN_3_ + *k*NH_4_F mixture under 2.0 MPa of nitrogen pressure. The entire combustion process, with the optimized NH_4_F amount used (4.0 mol), can be presented as follows:

(1) $$\begin{array}{c} K_{2}{\text{TaF}}_{7}+{9\text{NaN}}_{3}+{4\text{NH}}_{4}F\to 2\mathrm{KF}+9\text{NaF}\\ +\text{TaN}+{15N}_{2}+{8H}_{2}. \end{array}$$

As shown above, the forming of cubic TaN from the exothermic mixture of K_2_TaF_7_ + 5NaN_3_ composition does not occur despite a relatively high combustion temperature (1,170°C). Under conditions, however, the addition of ammonium fluoride to the reaction mixture had a favorable effect on the cubic-phase *δ*-TaN nanoparticle synthesis, despite large drops in the combustion temperature (850°C; *k* = 4). The replacement of NH_4_F with NH_4_Cl slightly lowered the combustion temperature to 850°C (*k* = 4). However, cubic-phase *δ*-TaN nanoparticles were obtained. Therefore, the addition of ammonium halides to the combustion reaction can provide low pressure and temperature route for the synthesis of the cubic TaN.

Ammonium halides appear to have two functions: acting first as a heat sink and then as a source of nitrogen and hydrogen. According to Equation 1, each mole of NH_4_F added to the mixture required 1.0 mol of NaN_3_ in order to neutralize HF, which forms after the decomposition of NH_4_F. Therefore, the intermediate gas phase products of the combustion process may consist of NH_3_, N_2_, and H_2_. However, at higher combustion temperatures (>500°C), a decomposition of NH_3_ occurs, and N_2_ and H_2_ gases become dominant. A simple estimation from Equation 1 shows that the total amounts of N_2_ and H_2_ in the combustion wave are 15.5 and 8 mol, respectively. We think that the presence of N_2_ and H_2_ gases in the combustion wave is the key factor, making cubic TaN formation favorable. In order to prove this assumption, we have prepared a hydrogen-free mixture of K_2_TaF_7_ + 5.175ZnF_2_ + 10.35 NaN_3_ composition and combusted under 2.0 MPa nitrogen pressure. The combustion process in the given system can be presented as follows:

(2) $$\begin{array}{c} K_{2}{\text{TaF}}_{7}+5.{175\text{ZnF}}_{2}+10.{35\;\text{NaN}}_{3}\to 2\text{KF}\\ +10.35\text{NaF}+\text{TaN}+{15N}_{2}+5.175\text{Zn}. \end{array}$$

In this process, the total amount of NaN_3_ was set at 10.35 mol to produce 15.5 mol of N_2_, as seen in the reaction (Equation 2). The combustion temperature of the K_2_TaF_7_ + 5.175ZnF_2_ + 10.35 NaN_3_ mixture measured by thermocouples was 900°C. The reaction product after acid leaching was a black powder and was a component from hexagonal *ε*-TaN and Ta_2_N according to XRD analysis. No peaks matching to cubic TaN was found on the XRD patterns. The response of cubic TaN to ammonium halides raised the question about the mechanism of the process. At present, we do not have a clear explanation of the role that ammonium halide has during the synthesis process. However, a plausible hypothesis can be offered with respect to the underlying mechanism. We believe that the hydrogen that is released from ammonium halide may stimulate a process of hydration-dehydration of Ta in the intermediate stages of the combustion process and may lead to vacancies in the tantalum lattice without affecting its crystal structure. These free vacancies created by hydrogen atoms could be easily occupied by nitrogen atoms at higher combustion temperatures, thus leading to the formation of cubic *δ*-TaN.

Another possible explanation for the cubic phase may involve the formation of tantalum amido- or imido-fluorides (Ta(NH_2_)_2_F_3_.4NH_3_ or Ta(NH_2_)_2_F_4_.6NH_3_) in a manner similar to the previously reported formation of tantalum amido- or imido-chlorides (Ta(NH_2_)_2_Cl_3_.4NH_3_ or Ta(NH_2_)_2_Cl_4_.6NH_3_) [18,19]. However a further, detailed investigation is needed to clarify the mechanism behind the formation of cubic tantalum nitride using ammonium halides.

## Conclusions

Nanocrystalline cubic δ-TaN was prepared by a solid combustion synthesis method using the K_2_TaF_7_ + (5 + *k*)NaN_3_ + *k*NH_4_F reactive mixture. It was shown that without NH_4_F, the maximum temperature of K_2_TaF_7_ + 5NaN_3_ mixture is 1,170°C, and the combustion product is multiphase consisting of hexagonal TaN as well as TaN_0.8_ and Ta_2_N phases. However, the addition of NH_4_F to the reactive mixture stimulates the formation of cubic *δ*-TaN. Phase-pure cubic *δ*-TaN was obtained when NH_4_F in the amount of 4.0 mol (or greater) was used in the combustion experiments. The formation temperatures for cubic *δ*-TaN were as low as 850°C to 950°C. Cubic *δ*-TaN synthesized using 4.0 mol of NH_4_F exhibited a specific surface area of 30.59 m^2^/g and a grain size of 5 to 10 nm, as estimated from its TEM micrograph. The approach developed in this study is a simple and cost-efficient method for the large-scale production of *δ*-TaN.

## Competing interests

The authors declare that they have no competing interests.

## Authors’ contributions

MHH, KHL, KSK, KKB, and JHL conceived the review. YJL performed the experiments with the help from DYK. YJL drafted the manuscript. All authors read and approved the final manuscript.

## Authors’ information

YJL is under the Ph.D. course in Green Energy Technology in Chungnam National University. DYK is under the master course in Green Energy Technology in Chungnam National University. KKB and KSK are principal researchers in Korea Institute of Energy Research. KHL and JHL are professors at the Graduate School of the Department of Metallurgical Engineering of Chungnam National University. MHH is a professor at the Graduate School of Green Energy Technology of Chungnam National University.
